# Influence of restorative material and cement on the stress distribution of endocrowns: 3D finite element analysis

**DOI:** 10.1186/s12903-021-01865-w

**Published:** 2021-10-05

**Authors:** Jiahui He, Ziting Zheng, Min Wu, Chunqing Zheng, Yuting Zeng, Wenjuan Yan

**Affiliations:** 1 Stomatology Health Care Center, Affiliated Shenzhen Maternity & Child Healthcare Hospital, Southern Medical University, Shenzhen, China; 2 Nanfang Hospital, Southern Medical University, Guangzhou, China

**Keywords:** 3D finite element analysis, Endocrown, Cement layer, Simulation of mastication, Endodontically treated teeth

## Abstract

**Purpose:**

This study aimed to evaluate the influence of different types of restorative materials and resin cements on the stress distribution in the regions of the restoration, cement layer and dental remnant in endodontically treated posterior endocrowns.

**Methods:**

A 3D finite element analysis (FEA) model of the first mandibular molar that was restored with an endocrown designed by computer-aided design (CAD) software was generated. Three kinds of restorative materials (Vita Enamic (VE), IPS e.max CAD (EMX) and Grandio blocs (GR)) and two types of cementing materials (NX3 and Maxcem Elite Chroma (MX)) were analysed with such a model. The food layer was also designed before vertical (600 N) forces were applied to simulate physiological masticatory conditions. Thermal expansion was used to simulate the polymerization shrinkage effects of cement layers. The results were obtained by colorimetric graphs of the maximum principal stress in the restoration and tooth remnant. The failure risk of the cement layer was also calculated based on the normal stress.

**Results:**

The elastic modulus was positively correlated with the tensile stress peak values in the restoration, mainly at the intaglio surface. However, in the cervical enamel and cement layer, restorative material with a higher elastic modulus generated lower peak stress values. The cement with a higher elastic modulus resulted in higher stress peak values inside the cement layer. The combination of EMX (restorative material) and NX3 (cement material) in the cement layer resulted in the lowest failure risk.

**Significance:**

The ceramic material EMX with a higher elastic modulus appeared to be more effective at protecting the cement layer and residual enamel tissue. Based on the analysis of the failure risk of the cement layer, the combination of EMX and NX3 was recommended as an optional material for endocrowns for endodontically treated posterior teeth.

## Introduction

In recent years, the development of adhesive dentistry, in conjunction with improvements in the mechanical properties of restorative materials and their manufacturing process, has enabled restorations to recover all or part of weakened tooth resistance, thus increasing the possibility of more conservative restorative procedures [1–3]. Less invasive preparations and greater preservation of tooth structure have been the new principles in teeth restoration [4]. The endocrown is a new minimally invasive restoration method for endodontically treated teeth (ETT), which is composed of a butt plane and retainer deeply fixed into the internal walls of the pulp chamber [5, 6], and the retentive effect benefits from the macroscopic and microscopic mechanical retentions provided by the pulp cavity and adhesion [7]. Endocrowns have proven superior to traditional full crown restorations in posterior teeth and superior to procedures documented in existing data on post- and core-based single crowns, and the survival rate is excellent at 99.0% after 44.7 ± 34.6 months [8–10].

Endocrowns can be made using computer-aided design/computer-aided manufacturing (CAD/CAM) technology, which minimizes clinical adjustment and defects during the manufacturing process and allows completed treatment in a single session. Recent studies have shown that the materials available for endocrowns mainly include resin composites [11], hybrid ceramics [12], lithium disilicate glass ceramics, zirconia reinforced lithium silicate [13] and feldspar ceramics. Each material has the ability to respond mechanically differently to the same applied masticatory force [14]. However, there have been no studies on the stress generated in the adhesive interface of endocrowns promoted by different materials. The stress generated at the adhesive interface is of interest to the dentist since the retentive effect of endocrowns is governed by adhesion and failure as reported in the literature regarding endocrowns, involving the detachment and marginal leakage of the adhesive margins [15–17]. Thus, a restorative material that optimizes the stress distribution in the adhesive interface during occlusal loading could alleviate the reported clinical problems and promote a higher endocrown success rate.

The reliability of adhesives is the key factor to achieve long-term satisfactory effects. Poor adhesion leads to micro-leakage, secondary caries and periodontal disease and ultimately repair failure [18]. The success of resin bonding depends on the bonding strength, mechanical strength, polymerization shrinkage, fatigue resistance and biocompatibility [19]. To achieve a satisfactory bonding performance between the resin cement and the substrate (prosthesis or tooth), several pre-treatment bonding steps are usually required [20]. However, this complicated procedure results in high technique sensitivity and a long chair time, which easily causes negative effects, such as contamination of the substrate with saliva or blood [21]. To simplify the adhesion procedures, self-adhesive resin cements were recently developed that can bond to the substrate without pre-treatment. The main components of the self-adhesive resin cement included the acid-functional monomer, dimethacrylate monomers (such as urethane dimethacrylate and Triethylene glycol dimethacrylate), filled granules and the activator-initiator system [22]. This eliminates the traditional pre-treatment of dentin surfaces, such as acid etching and rinsing, simplifies the clinical operation steps, creates micromechanical retention and chemical bonding, and provides greater moisture tolerance [23, 24]. However, some studies have shown that compared with traditional resin cement, self-adhesive resin cement has a lower adhesive strength to the tooth, possibly because the use of the acid monomer decreases the mechanical properties of the cement (flexural strength, hardness and wear resistance, etc.) to some extent [25, 26]. Whether different resin cement behaviours influence the longevity of the treatment is of interest for tooth restoration, and such an issue is also discussed in the present study.

To evaluate the stress distribution in ETTs generated by mastication, three-dimensional finite element analysis (3D FEA) has been widely used due to its low cost and standardized parameters [27]. Therefore, 3D FEA analysis was applied to evaluate the stress distribution at the regions of the restoration, cementing line and dental remnant structure, comparing the different restorative materials as well as resin cements that were used in the posterior endocrowns. The null hypothesis was that there was no difference in the stress distribution in the restorations and cementing line, regardless of the restorative materials and resin cements used.

## Materials and methods

**Fig. 1:**
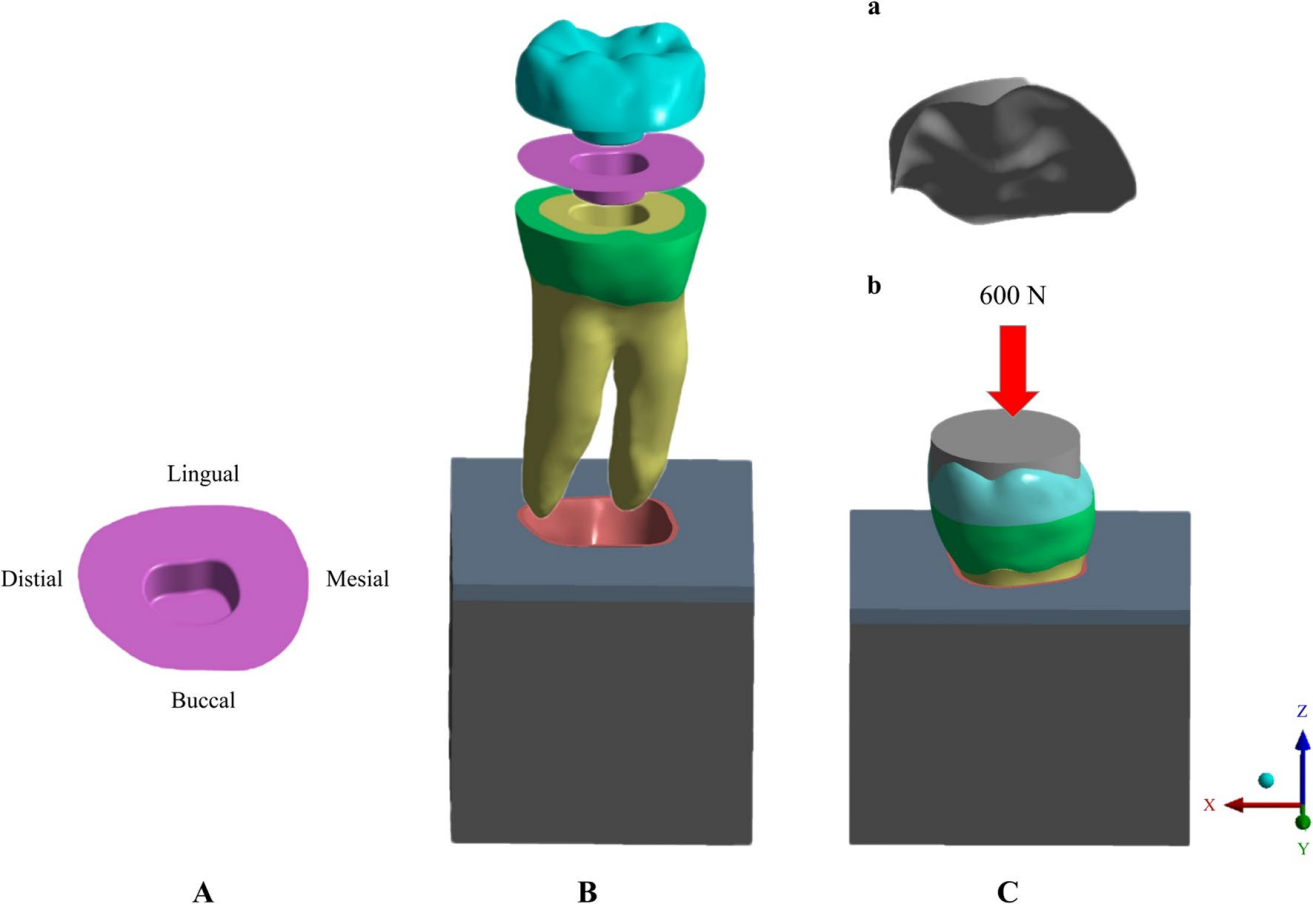
3D FEA model of an endocrown: (A) cementing line; (B) sketch of endocrown-restored molar; (C) a): food bolus on the occlusal surface; b) static load on the food bolus

A 3D CAD model of a healthy mandibular molar was built by means of a micro-computed tomography (micro-CT) scan system (Quantum GX; PerkinElmer) and CAD software (SolidWorks 2014; Dassault Systèmes) to generate the shapes of dentin, pulp and enamel. Starting from this 3D CAD model, endocrown models of flat design preparation and restoration with different materials were created. To simulate an ETT, the root canal was filled with gutta percha 1 mm below the orifice, and the pulp chamber floor was tiled with flowable resin (SDR; Dentsply Sirona). The endocrown was prepared as described in our previous study [28]: cuspal reduction of 2 mm, pulp chamber of 2-mm depth, and 8° wall inclination angle. The final geometries were monolithic endocrown, cement line, enamel, dental, periodontal ligament, flowable resin, gutta percha and alveolar bone (Fig. 1). The cement layer with 120-µm thickness was modelled by shell elements between the intaglio surfaces of the restoration and the bonding surfaces of the substrate. A food bolus [29] was modelled on the occlusal surface, and slide-type contact was used between the prosthesis surface and food (Fig. 1). The file of geometries was imported into FE software (ANSYS, v20.0; Swanson Analysis Inc.) as a polygon mesh composed of 345,467 nodes and 227,987 tetrahedral elements. Duplicates of the 3D model restored with an endocrown using 3 different CAD-CAM materials: Vita Enamic (VE, VITA Zahnfabrik), IPS e.max CAD EMX (EMX, Ivoclar-Vivadent AG) and Grandio Blocs (GR, VOCO) and 2 resin cements: resin composite cement (NX3, Kerr) and self-adhesive resin cement (Maxcem Elite Chroma, MX, Kerr).

The mechanical properties of the materials and structures used in this study were determined from published values (Table 1). All structures were assumed to be linear-elastic, isotropic, and homogeneously distributed. A linear thermal expansion coefficient was assigned to the cement layers to simulatr polymerization shrinkage effects (Table 1), assuming a one-degree drop in temperature, the cement layer shrinks and generates stress at the substrate-restoration interface [30]. To stimulate contact during the closing phase of the chewing cycle, an occlusal static load of 600 N and a bucco-lingual load of 20 N were uniformly applied on the surface of the food bolus [31, 32]. For all models, the maximum principal stress (MPS) on restoration and tooth remnant were evaluated in megapascals (MPa). For the cement layer, the normal stress perpendicular to the insertion trajectory (x-axis) was recorded [33, 34]. This is a non-failure condition in the analysis, and all the materials were assumed to be elastic materials throughout the entire deformation. Slide-type contact elements were used between the tooth surface and food and no-separation contacts were considered between restoration/resin cement and resin cement/tooth. For all other structures, the contacts were considered ideal. The boundary condition was the base of the alveolar bone with fixed zero nodal displacements. In addition, a formula was used to evaluate the between-group failure risk of the cement layer intuitively. For the cement layer, cohesive failure (fracture in cement layer) risks were calculated according to stress peak value divided tensile strength, while adhesive failure (damage at the interface between cement and substrate) risks were evaluated in terms of stress peak value to the adhesive bond strength to dentin ratio.

**Table 1:** Mechanical properties of materials and structures used in this study

Material	Modulus of elasticity (GPa)	Poisson’s ratio	Vol. shrinkage (%)	Linear thermal expansion coefficient	tensile strength (MPa)	Adhesive bond strength to dentin (MPa)
Food	10 [29]	0.3				
Dentin	18.6 [29]	0.31				
Enamel	84.10 [35]	0.33				
Core buildup resin	17 [35]	0.23				
Gutta percha	0.07 [3]	0.4				
Parodontium	0.69 [3]	0.45				
Cortical bone	13.7 [29]	0.3				
Cancellous bone	1.37 [29]	0.3				
Vita enamic (VE)	37.80 [3]	0.24				
IPS e.max CAD (EMX)	95.0 [3]	0.3				
Grandio blocs (GR)	18.0 [3]	0.26				
NX3	7.4^a^	0.35	4.88 [18]	0.0165	51.9^a^	33.8^a^
MaxCem Elite Chroma (MX)	4.0^a^	0.35	6.08 [18]	0.0207	46.5^a^	23.7^a^

^a^Supplied by the manufacturer

## Results

**Fig. 2:**
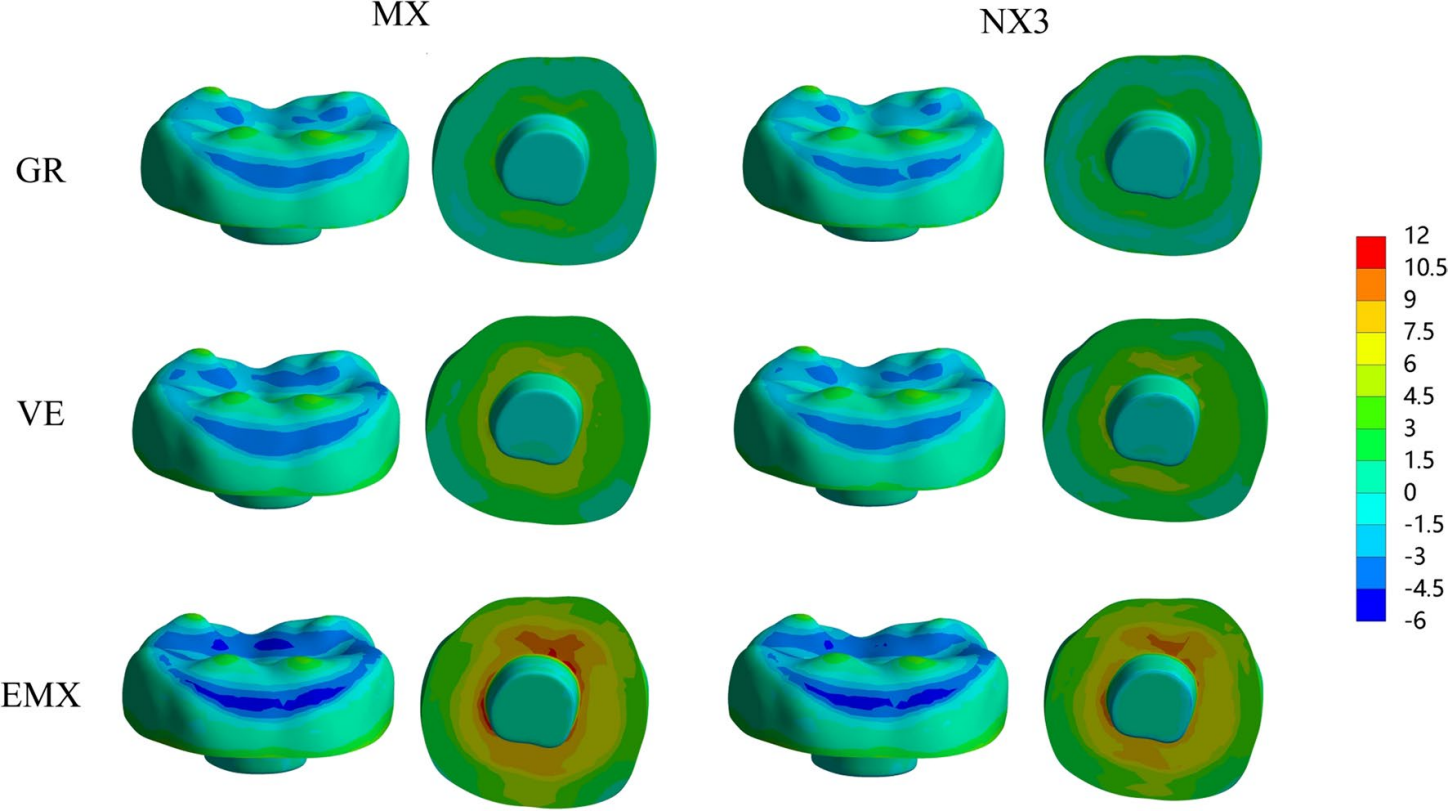
MPS generated in an endocrown: Restorations according to restorative material and resin cement. GR, Grandio blocs; VE, Vita Enamic; EMX, IPS e.max CAD; MX, Maxcem Elite Chroma

**Fig. 3:**
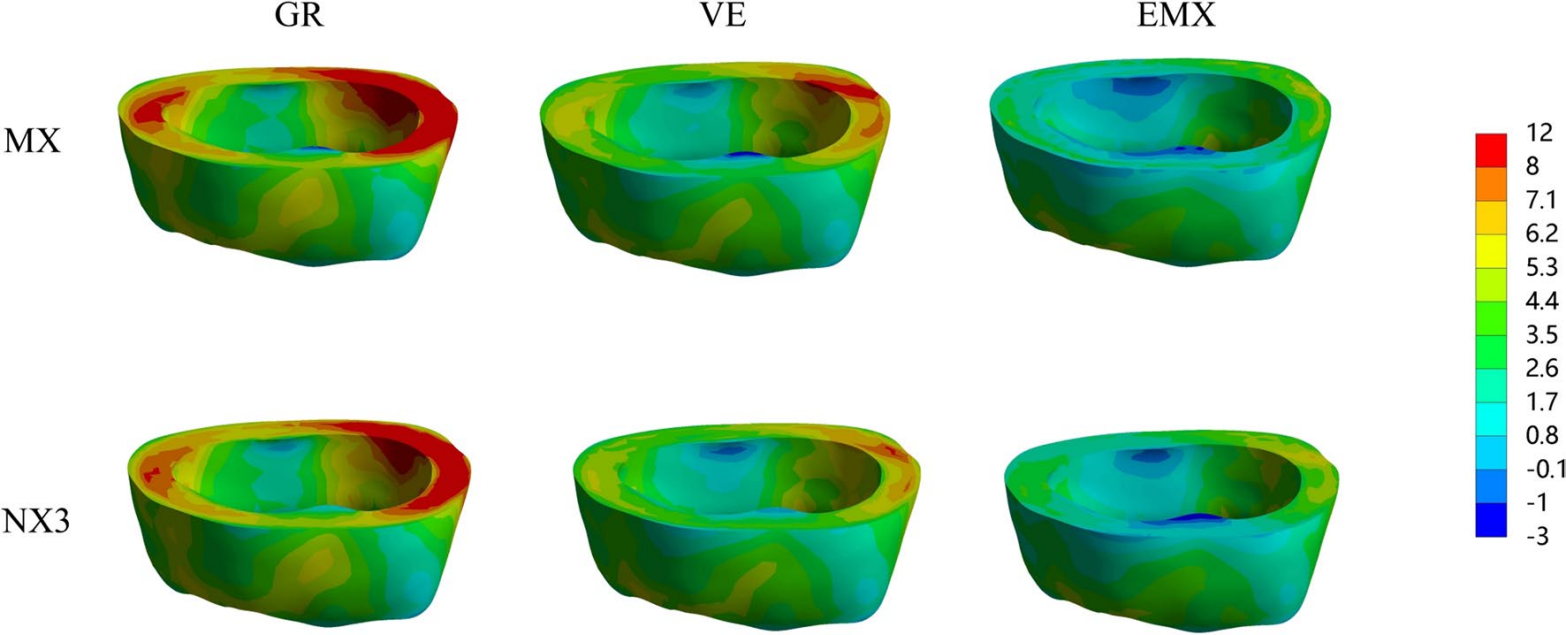
MPS generated in enamel according to the restorative material and resin cement type. GR, Grandio blocs; VE, Vita Enamic; EMX, IPS e.max CAD; MX, Maxcem Elite Chroma

**Fig. 4:**
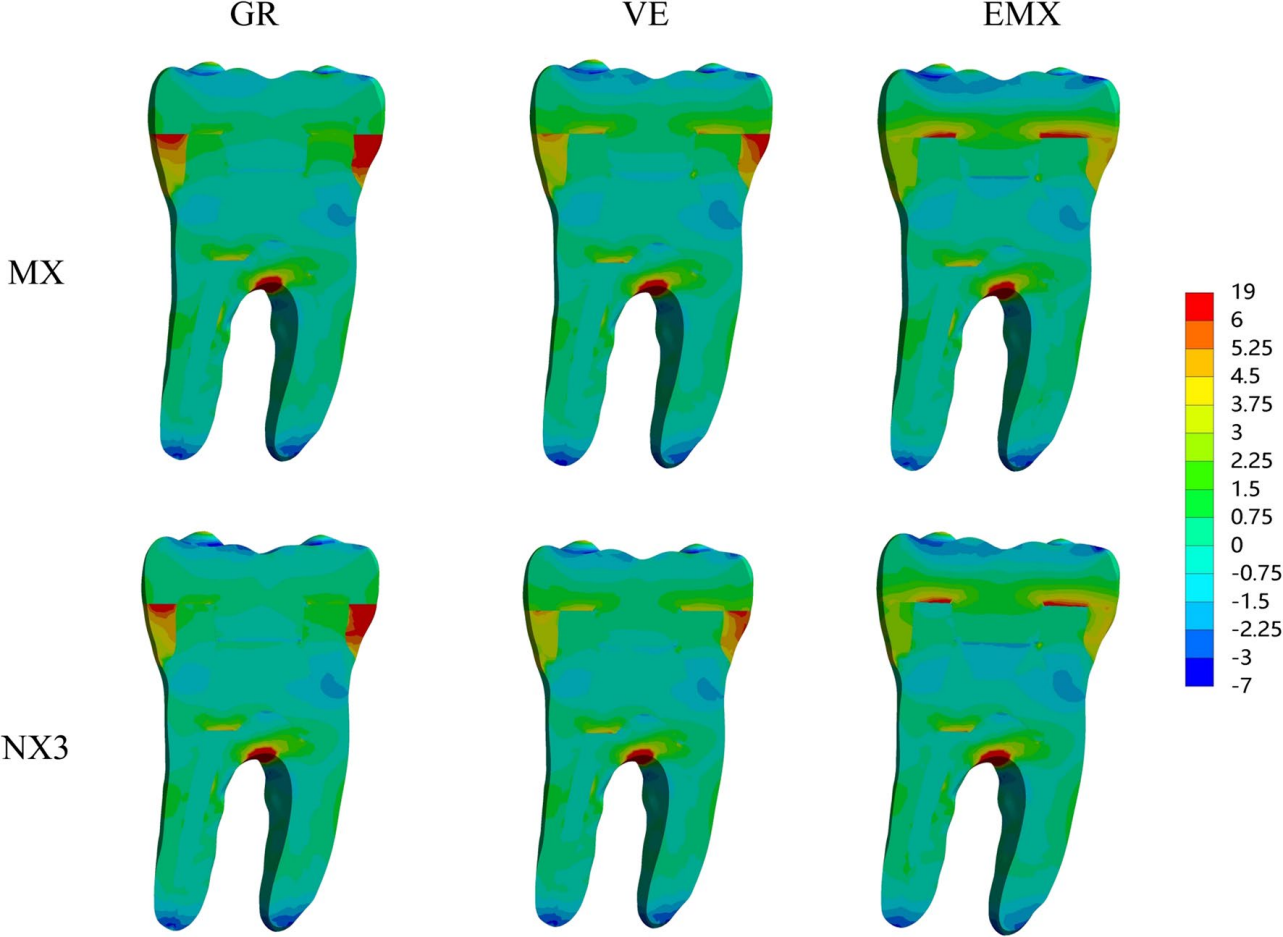
MPS generated in overall structures in the sagittal plane according to the restorative material and resin cement. GR, Grandio blocs; VE, Vita Enamic; EMX, IPS e.max CAD; MX, Maxcem Elite Chroma

**Fig. 5:**
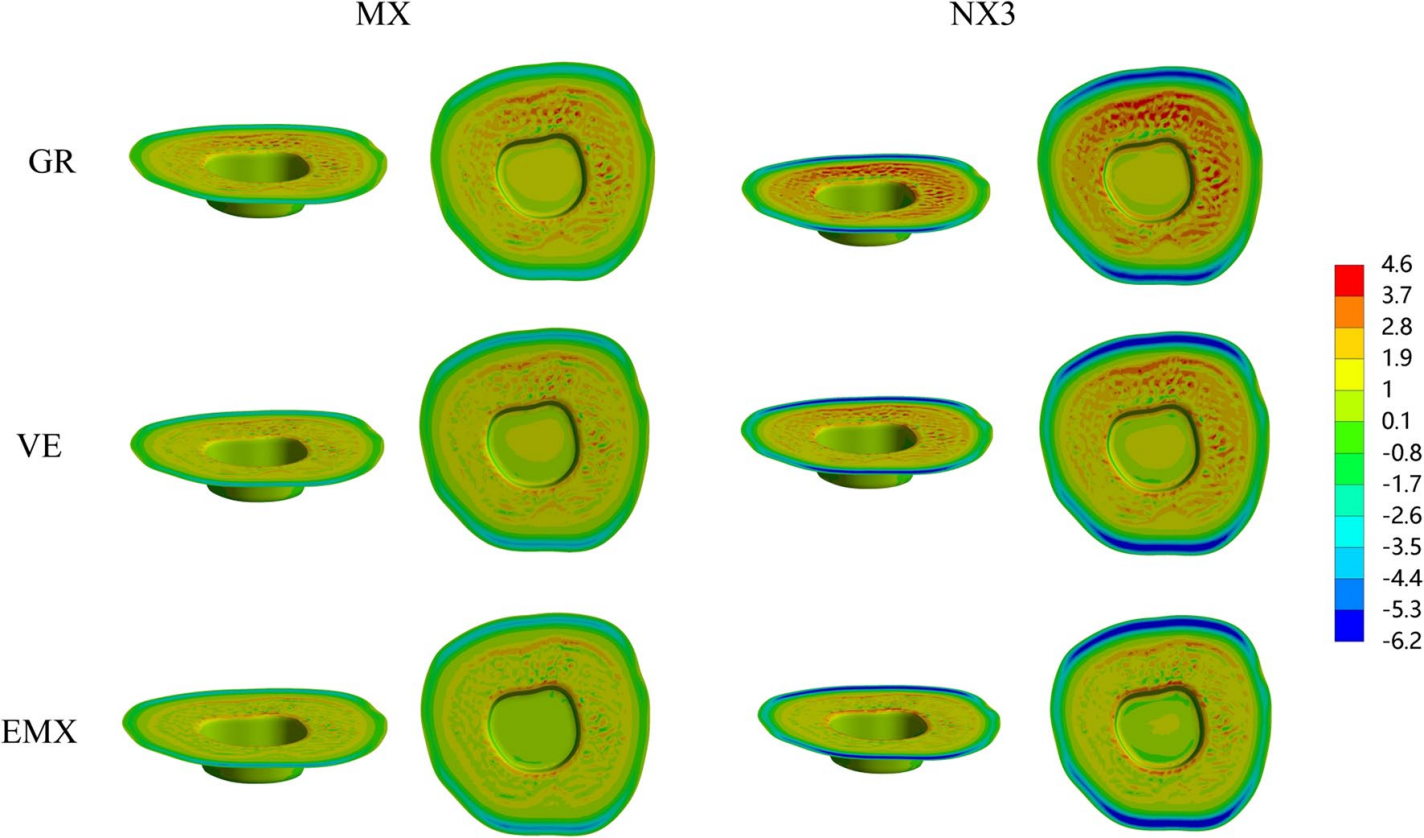
Normal stress generated on the cementing line according to the restorative material and resin cement. GR, Grandio blocs; VE, Vita Enamic; EMX, IPS e.max CAD; MX, Maxcem Elite Chroma

The colorimetric graphs in Figs. 2, 3 and 4 show the first principal stress distributions for enamel, dentin and restorative material in each model due to masticatory loads in combination with shrinkage effects. For the endocrown restoration, the tensile stress was concentrated mainly on the intaglio surface, especially the annulus area contact to dentin (Fig. 2). The stress distribution in the endocrown restoration was directly proportional to the elastic modulus of the restorative material, and a larger elastic modulus increased the tensile stress peak values in the restoration. Tensile stress in the remnants was concentrated in the distal and mesial sides of the cervical enamel (Fig. 3) and the furcation area of dentin (Fig. 4). From the view of cross section along the mesial-distal direction of overall structures (Fig. 4), the tensile stress was transferred from the enamel to the prosthesis with increasing elastic modulus of the restorative material. The stress peak values in the enamel (Table 2) revealed that GR-MX (11.64 MPa) had the highest stress value, 139% greater than the lowest value (EMX-NX3 at 4.88 MPa). The normal stress generated in the cement line (Fig. 5) was improved with increasing elastic modulus of the prosthetic material. In addition, cement with a higher elastic modulus also increased the stress peak values of the cement layer. In terms of the failure risk of the cement layer, the adhesive failure risk of all materials (0.104–0.170) was 70% higher than that of the cohesion (0.067–0.088), with the lowest risk for the EMX-NX3 group and the highest risk for group GR-MX (Table 3).

**Table 2:** Maximum principal stress values in restoration, enamel and dentin under loads (MPa)

Resin cement	Restorative material	Restoration	Enamel	Dentin
MX	GR	4.88	12.00	18.38
	VE	6.72	9.61	18.65
	EMX	11.64	8.94	18.81
NX3	GR	4.89	11.31	18.36
	VE	6.32	9.88	18.68
	EMX	10.57	9.19	18.87

**Table 3:** Normal stress peaks (MPa) and failure risks for cementing line

Resin cement	Restorative material		Stress peak	Failure risk		
				Cohesive Failure		Adhesive Failure
MX	GR		4.04	0.087		0.170
	VE		3.64	0.078		0.154
	EMX		3.16	0.068		0.133
NX3	GR		4.59	0.088		0.136
	VE		4.09	0.079		0.121
	EMX		3.5	0.067		0.104

## Discussion

This study aimed to evaluate the stressed regions in the posterior endocrown and in the cement layer according to different restorative materials and different resin cements. The results showed that the elastic modulus of the restorative material and the resin cement can influence the stress concentration at the adhesive interface. Thus, the hypothesis was rejected.

To accurately mimic the stress transmission during masticatory movement, the food layer was designed before vertical and horizontal forces were applied. Most 3D FEA studies for dental mechanical properties are carried out with three-point loading, resulting in the stress of the restoration always being concentrated on the loading point [3, 30]. In fact, the prosthesis covering dental cusps was assumed to be the greatest stress during the chewing cycle. Moreover, the stress on the occlusal surface of the prosthesis is regional rather than applied at an individual point [36, 37]. This explains why the fracture risk of restoration in finite element and Weibull analysis is much greater than those in practical tests [3].

From the colorimetric graphs, it is suggested that a higher elastic modulus of NX3 can reduce the peak stress in the prosthesis. However, the two kinds of cement had little effect on the peak value of the prosthesis, which might be the reason for the thin cement layer and the small discrepancy in the elastic modulus between NX3 and MX. Regarding the restorative material, the higher the elastic modulus was, the higher the stress concentration in the restoration. According to the manufacturer and previous studies [38], the biaxial flexural strengths of the IPS e. max CAD, VE and Grandio Blocs are 415 MPa, 174 MPa and 333 MPa, respectively, providing adequate capacity to withstand masticatory loads. In this study, EMX concentrated the maximum stress, which was far from reaching the fracture threshold, and the fracture risk of restoration was limited. However, the stress peaks can be harmful when located in the bonding surface of the restoration since this region is situated above the cement layer and dental remnant structure, and detachment of the restoration can be initiated in these stressed regions [38]. One study suggested that excessive variation in the elastic modulus between the prosthesis and the substrate may cause stress concentration between the interface, degrade the cement layer and increase the fatigue mechanisms inside it [39]. Similar to a study concerning the full crown [39], the resinous material GR, with an elastic modulus more consistent with dentin, dissipated more energy and decreased the stress variation between the dentin and endocrown (Fig. 4). However, under the same load, more stress variation was present between the GR endocrown and enamel, in contrast to EMX, where the stress was more uniformly distributed and not concentrated at the interface. Clinically, the marginal cement between restorations and enamel has a high probability of direct exposure to the oral environment. When adhesive failure occurs, it can cause secondary caries, pulpitis, and even fracture of the restoration [40], thus affecting the adhesive effect of endocrown-restored mandibular molars. Therefore, the EMX endocrown seems to be more effective in protecting the interface integrity and improving long-term effectiveness.

It is notable that some studies [39, 41] did not simulate the cement layer, and the analysis of the difference in elastic modulus does not seem to explain the stress accumulation of the cement layer. Since failures on the cement layer can occur in the long term and cause restoration displacement, there may be associated research value. According to previous studies [42, 43] and our results, the presence of the cement line may affect the system mechanical distribution, so the cement layer should be modelled as an individual structure to analyse the adhesive and cohesion failure of bonding.

Referring to Fig. 5, it is possible to note that the more rigid the restoration, the less stress reaches the cement. Similar to previous studies, restorations with a higher elastic modulus concentrated more stress and exhibit markedly smaller deformation [44]. Thus, the peak stress value in the cement layer decreased, meaning that the use of a high-elastic-modulus material benefits bonding. Moreover, MX resulted in a lower stress distribution in the cement layer, which suggests that considering only the single variable of the elastic modulus, low-elastic-modulus cement materials theoretically decrease the risk of bonding failure. It has been observed that mechanical properties (bonding strength with matrix, tensile strength, etc.) can vary widely between self-adhesive resin (MX) cements and conventional resin cements (NX3) [25], and the failure risk of bonding is also affected; hence, we used the formula to calculate the failure risk. Benefitting from the superior bonding performance and tensile strength, the results of the failure calculation showed that NX3 has a lower bonding failure risk (including cohesive failure and adhesive failure) than MX. In addition, contrary to previous research results [30, 33], our results showed that the failure risk of bonding is generally higher than the failure risk of cohesion of approximately 70%. According to the formulas, the mechanical properties of resin cement directly affect the risk of cohesive and adhesive failure. Therefore, this result may be due to due to the higher tensile strength of the selected resin cements (Maxcem, 46.5 MPa; NX3, 51.9 MPa) in this study compared to the resin cements (approximately 14 MPa) in previous study [33].

Regarding the dental remnant structure, higher stress peaks concentrated in the surrounding enamel (in particular, mesial and distal margins), which was the initial area where failure occurred, were observed in the materials with a lower elastic modulus (VE or GR). Due to such a stress distribution, fracture may occur in the enamel edge and propagate to the inner enamel, resulting in micro-leakage around the affected restoration [45]. It is known that enamel, with greater bond strength and higher elastic modulus among dental tissues, tends to concentrate more stress whenever it is present, and its integrity directly influences restoration longevity [46–48]. Therefore, compared to GR and VE, the ceramic material EMX appears to be more effective at protecting the residual enamel tissue, corroborating previous in vitro studies [49].

When incorporating the analysis of stress distribution for overall structures and the failure risk assessment for the cement layer, despite all limitations of this study, the group restored with EMX and NX3 is recommended regarding endocrowns for endodontically treated posterior teeth. The results of in vitro simulation testing cannot be predictively extended to the clinical situation because this simulation study design did not consider typical factors, such as wear resistance, fatigue resistance or aesthetics. This study only analysed the stress distribution of endocrown molars under a static load at closing phase of the chewing cycle. Therefore, future studies with dynamic loading and clinical trials with long follow-up periods are indicated.

## Abbreviations

3D: Three dimensional
FEA: Finite element analysis
CAD: Computer-aided design
CAM: Computer-aided manufacturing
VE: Vita Enamic
EMX: IPS e.max CAD
GR: Grandio blocs
MX: Maxcem Elite Chroma
N: Newton
ETT: Endodontically treated teeth
MPS: Maximum principal stress
MPa: Megapascals
